# Correspondence: Numerical modelling of the PERM anomaly and the Emeishan large igneous province

**DOI:** 10.1038/s41467-017-00125-2

**Published:** 2017-10-10

**Authors:** Trond H. Torsvik, Mathew Domeier

**Affiliations:** 1 Centre for Earth Evolution and Dynamics (CEED), University of Oslo, 0315 Oslo, Norway; 2 Helmholtz Centre Potsdam, GFZ, Telegrafenberg, 14473 Potsdam, Germany; 3 NGU Geodynamics, 7040 Trondheim, Norway; 4 School of Geosciences, University of Witwatersrand, Johannesburg, 2050 South Africa

## Introduction

Large igneous provinces (LIPs) are the result of catastrophic melting in the upper mantle, and by reconstructing their positions over the past 300 Myr it has been shown that most LIPs—including the 260 Myrs old Emeishan LIP in South China—probably originated from plumes at the edges of two large low-velocity regions in the lowermost mantle. In a recent article published in *Nature Communications*, Flament et al.^1^ presented a remarkable new view on the origin of the Emeishan LIP (based on numerical modelling) and we discuss here why we do not agree with their interpretation.

**Fig. 1:**
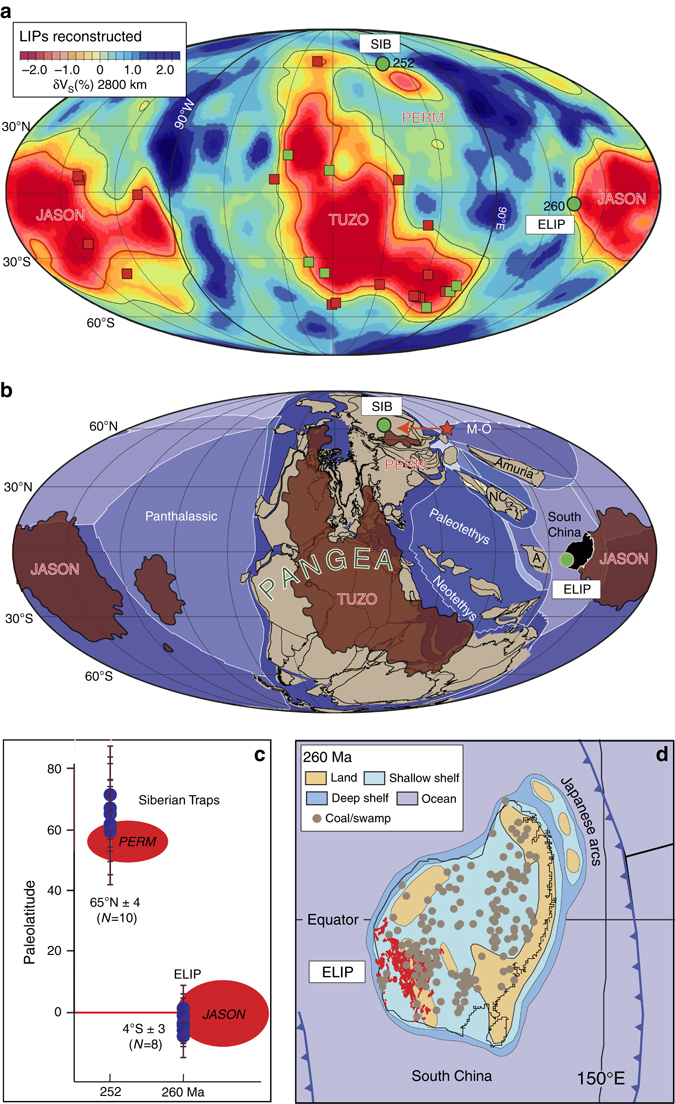
Reconstruction of large igneous provinces. **a** Reconstruction of 26 large igneous provinces (*LIPs*, 31–297 Ma) using a hybrid reference frame^9^ and draped on the s10 mean tomographic model^10^. The plume generation zone (*PGZ*, *thick red line*) in this model corresponds to the 0.9% slow contour and the zero contours are shown as *thinner black lines*. LIPs with *red symbols* reconstructed with moving and fixed hotspot reference frames, while those with *green symbols* use a true polar wander-corrected palaeomagnetic reference frame. But the reconstruction of the 260 Ma Emeishan LIP (*ELIP*) is an exception: Although Pangea was amalgamated at 260 Ma (**b**), the supercontinent did not include South China, which is therefore without longitudinal constraints. The ELIP is on the South China block, and palaeomagnetic results position it at latitudes around 4° S (**c**); if ELIP had erupted above a PGZ, there are several possible longitudinal locations where the line of latitude crossed the PGZ at that time. Pangea covered TUZO (**b**), leaving only the options related to JASON, and the reconstruction with ELIP above the western margin of Jason at ~134° E, is a realistic alternative. One should also note that net true polar wander was zero between 250 and 260 Ma^11^. **b** Pangea reconstruction at 260 Ma^7, 12^ with plate boundaries and draped on seismic voting-map contour 4 in the lower mantle^6^. Here we only show the reconstructed location of the Siberian Traps (*SIB*, erupting ~8 million years after the reconstruction) and the ELIP that erupted at the equator and linked to the margin of JASON. The *red star* is where Flament et al.^1^ initiate the PERM anomaly at 190 Ma (linked to the much older ELIP!) and later shifted westwards to its current location. *A* Annamia (Indo-China), *M-O* Mongol-Okhotsk Ocean, *NC* North China. **c** Palaeomagnetically derived palaeolatitudes from the Siberian Traps (10 studies) and Emeishan volcanics (*ELIP*, 8 studies) with 95% confidence bars which form the basis for reconstructing South China at that time^11, 12^. **d** Detailed 260 Ma reconstruction of South China with plate boundaries^7^ and draped with Guadalupian (272–260 Ma) and Lopingian (260–252 Ma) coal/swamp occurrences^13^ that verify tropical (equatorial) humid conditions during the eruption of ELIP

The fundamental observation that most LIPs—when reconstructed—overlie the edges of two low-velocity regions near the core-mantle boundary (Fig. 1a) was first reported by Burke & Torsvik^2^. Two equatorial and antipodal regions argued to be the most probable sources of the mantle plumes that generated the LIPs were dubbed large low shear-wave velocity provinces (LLSVPs) by Garnero et al.^3^ and later TUZO (beneath Africa) and JASON (beneath the Pacific) by Burke^4^. Burke and co-authors argue that plumes mainly form at the margins of these LLSVPs, which have remained approximately stable through time. It was also noted, more than 10 years ago^5^ that the Siberian Traps (~252 Ma) when reconstructed overlie a smaller anomaly in the lower mantle which appears as a north-eastern arm of TUZO in many tomographic models (Fig. 1a), but is argued to represent a separate anomaly by Lekic et al.^6^ This anomaly was dubbed PERM and appears more isolated when using seismic voting-maps; as an example, we show voting-map contour 4^6^ in Fig. 1b, i.e. four seismic models show slower than average velocities in the lower mantle (1000–2800 km) beneath these regions.

As the long-term stability of the LLSVPs has broad implications for geodynamics, tectonics and palaeogeography, it is natural and necessary to formulate new questions and methods to evaluate this hypothesis. We thus read with great interest the recent article of Flament et al.^1^, wherein it is claimed that the PERM anomaly is both young (less than 200 Myr old) and highly mobile (having travelled ~1500 km westward in the last 150 Myr), and was linked to the Emeishan LIP (ELIP) in contrast to the Siberian Traps—assertions which, according to the authors ‘challenges the current understanding of the evolution of the plate–mantle system in which plumes rise from the edges of the two LLSVPs, spatially fixed in time’.

However, despite that strong statement by the authors^1^, we find their analysis flawed and thus question the validity of their findings. We begin by noting that, while the ELIP was formed at ~260 Ma (as acknowledged by the authors) and the Siberian Traps formed at ~252 Ma, the global mantle flow models of Flament et al.^1^ started at 230 Ma, or ~20–30 Myr after the eruption of the LIPs of interest. Even ignoring the two-way mantle transit time (the time for a slab to sink from the surface to the core-mantle boundary plus the time for a plume to make the return trip), the fact that the modelled time interval post-dates the eruption of the LIPs means their causality cannot be meaningfully interpreted from the model results. As an aside, there are already plate reconstructions with dynamic plate-polygons available back to 410 Ma^7^, which would allow global mantle flow models to be conducted in a more appropriate context of time to consider the formation of the Siberian Traps and ELIP. Because the authors chose not to employ a Palaeozoic plate model, they instead inserted slabs directly into the lower mantle at locations prescribed by their 230 Ma tectonic reconstruction, presupposing that the location of subduction did not change for 50–100 Myr. Furthermore, the authors^1^ assertion that PERM was generated at ~190 Ma implies that the anomaly was generated ~70 Myr after the LIP it is claimed to have produced. We also consider it important to highlight that the ELIP—which the authors link to the PERM anomaly—has been repeatedly shown by palaeomagnetic studies to have formed at equatorial latitudes (4 ± 3° S; Fig. 1c, d), whereas PERM, according to the reported results of the mantle flow models, formed at 60° N (the authors also wrote ‘100° W’, but we assume they meant ‘100° E’). In other words, rather than the Siberian Traps, which palaeomagnetic studies confirm did form near 60° N (65 ± 4° N; Fig. 1c) the authors prefer to link PERM with a LIP that was formed 60° away, at the equator.

This leads us to the question: if the Siberian Traps, when reconstructed, plot directly above the present PERM anomaly (Fig. 1a–c), and even the mobile anomaly modelled by Flament et al.^1^ appeared at this same latitude (as the Siberian Traps), why did the authors instead prefer a link with a LIP which formed at the equator? We suspect that the authors^1^ recognised that mobility observed among the lower mantle structures produced by their mantle flow models was not sufficient to draw a parallel inference about the real-world stability of the LLSVPs. Indeed, the visual resemblance of the lower mantle structures produced by their models (from an initially uniform basal layer) with those seismically imaged (in the present day) does not itself validate the notion that mantle flow can strongly (re)shape lower mantle structure over relatively short timescales (>200 Myr), as such an outcome can be equally well understood to confirm that the plate system obeys an already established lower mantle structure that guides subduction at the surface^8^. As pure modelling cannot break this causality dilemma, it is necessary to look to the geological record to test and substantiate model predictions, and here we consider the strong correlation between the margins of the present day LLSVPs and the reconstructed location of LIP eruptions of the last 300 Myr to present a compelling case for LLSVP stability (Fig. 1a). Despite the accordingly conspicuous reconstruction of the Siberian Traps directly above the present-day position of PERM, Flament et al.^1^ have tried to draw a link to ELIP in order to substantiate the mobility of PERM observed in their flow models—but the basis presented for such a link is untenable. Although we greatly look forward to future discussions and debate regarding the long-term stability of the LLSVPs, and value the insights that have been offered through modelling, as well as those yet to come, we hope this brief correspondence can serve as a reminder to be prudent with model validation.
